# Intragenomic diversity of *Rhizobium leguminosarum *bv. *trifolii *clover nodule isolates

**DOI:** 10.1186/1471-2180-11-123

**Published:** 2011-05-30

**Authors:** Andrzej Mazur, Grażyna Stasiak, Jerzy Wielbo, Agnieszka Kubik-Komar, Monika Marek-Kozaczuk, Anna Skorupska

**Affiliations:** 1 Department of Genetics and Microbiology, Maria Curie-Skłodowska University, Akademicka 19, 20-033 Lublin, Poland; 2 Chair of Applied Mathematics and Informatics, Lublin University of Life Sciences, Akademicka 13, 20-950 Lublin, Poland

## Abstract

**Background:**

Soil bacteria from the genus *Rhizobium *are characterized by a complex genomic architecture comprising chromosome and large plasmids. Genes responsible for symbiotic interactions with legumes are usually located on one of the plasmids, named the symbiotic plasmid (pSym). The plasmids have a great impact not only on the metabolic potential of rhizobia but also underlie genome rearrangements and plasticity.

**Results:**

Here, we analyzed the distribution and sequence variability of markers located on chromosomes and extrachromosomal replicons of *Rhizobium leguminosarum *bv. *trifolii *strains originating from nodules of clover grown in the same site in cultivated soil. First, on the basis of sequence similarity of *repA *and *repC *replication genes to the respective counterparts of chromids reported in *R. leguminosarum *bv. *viciae *3841 and *R. etli *CFN42, chromid-like replicons were distinguished from the pool of plasmids of the nodule isolates studied. Next, variability of the gene content was analyzed in the different genome compartments, i.e., the chromosome, chromid-like and 'other plasmids'. The stable and unstable chromosomal and plasmid genes were detected on the basis of hybridization data. Displacement of a few unstable genes between the chromosome, chromid-like and 'other plasmids', as well as loss of some markers was observed in the sampled strains. Analyses of chosen gene sequences allowed estimation of the degree of their adaptation to the three genome compartments as well as to the host.

**Conclusions:**

Our results showed that differences in distribution and sequence divergence of plasmid and chromosomal genes can be detected even within a small group of clover nodule isolates recovered from clovers grown at the same site. Substantial divergence of genome organization could be detected especially taking into account the content of extrachromosomal DNA. Despite the high variability concerning the number and size of plasmids among the studied strains, conservation of the location as well as dynamic distribution of the individual genes (especially replication genes) of a particular genome compartment were demonstrated. The sequence divergence of particular genes may be affected by their location in the given genome compartment. The 'other plasmid' genes are less adapted to the host genome than the chromosome and chromid-like genes.

## Background

Rhizobia are widely occurring soil bacteria that are able to establish nitrogen-fixing symbioses with legumes. Bacterium-plant interaction is a complex process in which specific plant and bacterial signals are exchanged resulting in formation of nodules, where rhizobia in the form of bacteroids fix nitrogen [1-3].

Rhizobial genomes are large and multipartite, composed of a single circular chromosome and a set of large plasmids [4-6]. The genes responsible for nodulation (*nod*) and nitrogen-fixation (*nif-fix*) are either carried by large plasmids (pSym) or are incorporated in the chromosome as symbiotic islands [7,8]. Large genomes of *Rhizobiaceae *and *Bradyrhizobiaceae *(above 6-9 Mb) are considered more ecologically advantageous in an environment that is scarce in nutrients but diverse as regards carbon and energy sources. These genomes are disproportionately enriched in regulation and transport genes and in genes involved in secondary metabolism in comparison with medium-and small-size genome containing bacteria [9].

"Core" and "accessory" components of *Rhizobium *genomes can be distinguished. Chromosomes with conserved gene content and order (synteny) are considered as core. Accordingly, plasmids constitute the accessory genome. Plasmids are more flexible than the chromosomes, as defined by more frequent gene gains and losses, even in the same species. They are heterogeneous in size and gene content and lack synteny even in closely related species, except for genes involved in plasmid replication and symbiotic properties [6,10,11]. In some species, such as *Rhizobium leguminosarum*, plasmids may comprise up to 35% of the total genome [6,7].

Rhizobial plasmids are maintained in the cells via *repABC *cassettes, comprising genes required for active segregation (*repAB*) and initiation of replication (*repC*) [12]. The presence of several *repABC *operons within a single genome, which are subjected to individual selection pressure and divergence, could be the key element of the existence of different plasmid incompatibility groups in cells and could drive the rearrangement of gene organization and of their functions [11,13-15]. It was proposed that *repABC *plasmids coexisting in the same strain most probably emerged by separate events of lateral transfer, which required evolution of different incompatibility groups allowing simultaneous residence of plasmids equipped with a similar replication/partition system in a single bacterial species [12]. Thus, the degree of divergence of the plasmid replication apparatus, whose sequence is subject to strong evolutionary pressure and determines the ability to evade incompatibility between plasmids [13], and horizontal gene transfers are potential forces that shaped rhizobial genomes.

Recently, some (not only rhizobial) extrachromosomal replicons that have properties distinct from both chromosome and plasmids were reported and named "chromids" [16]. Chromids are characterized by presence of some important genes essential for growth under all conditions, with nucleotide composition and codon usage similar to the chromosome of the parental strain, and, by contrast, plasmid replication and partition systems [16].

Furthermore, recent analyses of *Rhizobium etli *strains [11] showed that this species has a pangenomic structure. By definition, a pangenome "determines the core genome, which consists of genes shared by all the strains studied and probably encoding functions related to the basic biology and phenotypes of the species" [17]. The basis of the pangenome concept emerged from an observation that each newly sequenced genome enriched the pool of species-specific genes with new ones [17,18]. This makes it possible to detect, besides the core genomes, the dispensable genomes composed of both chromosomal and plasmid genes, present only in some of the strains, which contribute to the species diversity and allow adaptation to new ecological niches and a specific environment. Despite the overall genomic divergence, *R. etli *pangenome comprises a core genome composed of both chromosomal and plasmid sequences, as well as highly conserved symbiosis-related genes on the pSym plasmid. The unusual variability observed in rhizobial genomes may further result from several types of alterations, such as point mutations, deletions, amplification of DNA, and from intragenome re-assortment of sequences [19-21].

The aim of this study was to evaluate the divergence of genomes of a small population of *R. leguminosarum *bv. *trifolii *(*Rlt*) nodule isolates from clover plants grown in the same site in cultivated soil. Like the other members of the genus *Rhizobium*, the *Rlt *genomes were partitioned into the chromosome and several large plasmids, one of which carried symbiosis-related genes. The variability of the genome architecture involved not only the number and size of the plasmids, but also the location of specific genes on the particular replicons. Distribution of *repABC *operon markers and other genes in the three genome compartments: the chromosome, chromid-like and 'other plasmids' was assessed. We found "stable" genes that were permanently located in a specific genome compartment, as well as "unstable" ones, which were detected in different replicons of the sampled strains. Sequences of selected chromosome and plasmid genes were subjected to an assessment of adaptation to a particular genome compartment by analyses of codon usage and codon adaptation index. A potential evolutionary pathway of *Rlt *strains was proposed on the basis of gene sequences and their distribution.

## Methods

### *R. leguminosarum *bv. *trifolii (Rlt) *strains

129 *R. leguminosarum *isolates were obtained from nodules of red clover (*Trifolium pratense *L. cv. Dajana) growing in sandy loam (N:P:K 0.157:0.014:0.013%). Plants were grown on 1 m^2 ^plot for six weeks between May and June 2008. Afterwards, ten randomly chosen clover plants growing in each other's vicinity were harvested, the nodules were collected, surface-sterilized, crushed and their content plated on 79CA medium [22]. Strains isolated from the nodules were purified by successive streaking of single colonies and pure cultures were used in further experiments.

### DNA methods

Standard techniques were used for labeling of DNA, Southern hybridization and agarose gel electrophoresis [23]. DNA probes for Southern hybridizations were obtained by PCR amplification with *Rt*TA1 genomic DNA as template and appropriate primers (Table 1). The probes were labeled with non-radioactive DIG DNA Labeling and Detection Kit (Roche). Southern blotting, gel pretreatment and capillary transfers were done using standard procedures [23]. Hybridizations were performed at high stringency at 42°C using 50% formamide in pre-hybridization and hybridization solutions. Analyses of the plasmid content of the 129 isolates were performed as described by Eckhardt [24].

**Table 1:** Primers and probes used in this study

*Rt*TA1 replicon name	Probe name	Probe description	Primers	GenBank accession no
ch^a^	*pssL* (Pss-I)	1300 bp of Pss-I region encoding part of putative flippase PssL	pssLFw 5'-TCTTATCCGCCACGAATCTCC-3' pssLRw 5'-GCCAGGTGAAGGGCCAGCGCCAACT-3'	DQ384110
	*rfbADBC* (Pss-V)	2956 bp of Pss-V region encoding lipopolysaccharide biosynthesis proteins	rfbAFw 5'-TCGAGATAGGTGCGGTTGACGTCG-3' rfbCRw 5'-GCAGGGCAACGCTGGTGCGCTGC-3'	DQ679959
	*bioA*	445 bp fragment encoding adenosylmethionine-8-amino-7-oxononanoate aminotransferase	bioA3 5'-CCTCGTCGAAGATCAGAAGG-3' bioA5 5'-TCTACACCAACTCCGGTTCC-3'	DQ535896
	*rpoH2*	487 bp fragment encoding RNA polymerase sigma factor	rpoH2Fw 5'-TGGTGCAGGAGGGCTATGTT-3' rpoH2Rw 5'-TCGCGTTCGTTGAGATGTTTC-3'	DQ366597
	*dnaC*	624 bp fragment encoding DNA helicase	dnaCFw 5'-CAGCCCGGCATTTTCACC-3' dnaCRw 5'-CTGCGGCCGTTTATCGTC-3'	DQ855524
	*dnaK*	645 bp fragment encoding heat shock protein 70 family	dnaKFw1 5'-CGTCATCACCGTTCCCGCCTACTT-3' dnaKRw1 5'-TTGCCGAACAGCTGCTTGACGACT-3'	DQ535895
	*exoR*	416 bp fragment encoding negative regulator of exopolysaccharide synthesis	exoRFw 5'-GTTGCCGCCTGCCTGAGATGAAC-3' exoRRw 5'-GAGAGCAGCGCGTTGACGAAGAAG-3'	DQ347956
	*rrl*	1135 bp fragment comprising rRNA genes *rrl *and *rrs-rrl *intergenic spacer	FGPS1490 5'-TGCGGCTGGATCACCTCCTT-3' FGPL132 5'-CCGGGTTTCCCCATTCGG-3'	DQ639765
	*lpxQ*	850 bp fragment encoding lipid A oxidase	lpxQFw 5'-GACGGCAAATTTCAGCGGCACATA-3' lpxQRw 5'-GGCGGCTGAGCAACACTTACCAA-3'	DQ836933
	*stbB*	423 bp fragment encoding plasmid stability protein	stbBFw1 5'-ATGATCGTTCTCGATACGAATGTGATTTC-3' stbBRw1 5'-TCAGCCGTCTTCAAACGGGTTT-3'	FJ230890
	*fixGH*	539 bp fragment encoding nitrogen fixation cation transport proteins	fix2Fw 5'-GCGGATTTCGTGCCCCTTTATGGA-3' fix2Rw 5'-TCTCTGCGGAATGGCTACACG-3'	DQ314612

pRleTA1d	*prc*	442 bp fragment encoding C-terminal tail-specific protease precursor	prcFw 5'-CGGCTTGCGCTTTTGTAATCCTG-3' prcRw 5'-CGCTGTTTGGTATCGGTGCTGTGC-3'	EF107512
	*hlyD*	620 bp fragment encoding type I secretion membrane fusion	hlyDFw 5'-CGAAGTCCGCGCCCGTGTG-3' hlyDRw 5'-TCGCGACCTTGACCTTGATGG-3'	EF123039
	*repAd*	1467 bp fragment encoding putative replication/partition protein of pRleTA1d	repAdFw 5'-CGCCGTGCCGCCATTTGA-3' repAdRw 5'-GGACTCCAGAGCCCGATCGTAGGTTC-3'	FJ592234
	*repCd*	578 bp fragment encoding putative replication protein of pRleTA1d	repCdFw 5'-CCGGACGAGCAAAGACTGAAACAA-3' repCdRw 5'-GACCGAGAGCCCGAATTTTTGTGT-3'	FJ592234

pRleTA1c	*lpsB2*	740 bp fragment encoding dTDP-glucose 4,6-dehydratase, O-antigen biosynthesis protein	lpsBFw 5'-GCACATCCGTAAAGCCAGGGTCAA-3' lpsBRw 5'-GCGGGTTATATCAGGATGTGTCAG-3'	DQ677348
	*orf16*, *orf17*, *otsB*	2191 bp fragment encoding component of ABC transporter Orf16 of AraC family, transcriptional regulator Orf17, trehalose-phosphatase OtsB	orf16Fw 5'-AAGCTTCTTCATTTCCGCGACGAAGCCC-3' ostBRw 5'-AAGCTTGGCGGTGCCTTGGCACTG-3'	FJ237527
	*tauA*-*orf14*	5 kb fragment encoding taurine uptake protein TauA and flavin monooxygenase/reductase Orf14 protein	tauAFw 5'-CCCGAGATGGAGGCGAGGTAAAAG-3' orf14Rw 5'-TTGGCAAGGCAGACGAGGAGAAG-3'	ED797712 ED797713
	*repAc*	433 bp fragment encoding replication/partition protein of pRleTA1c	repAcFw 5'-GATTTGCGTGAAYGYCGACCA-3' repAcRw 5'-AGGTGGATTGATGTCGTCGTCTTG-3'	EU555187
	*repCc*	1417 bp fragment encoding replication protein of pRleTA1c	repCcFw 5'-AGTTTTTGGCGCCGTTTTGGTGAG-3' repCcRw 5'-TATCTGACCGAGGCTGCTAACCAC-3'	EU555187

pRleTA1b	*pssM* (Pss-III)	440 bp fragment encoding surface polysaccharide biosynthesis protein of Pss-III region	MnewFw 5'-CGCAACACCGGGATTTCTG-3' MnewRw 5'-TCGGCGGGTATGGCGTGAT-3'	DQ417329
	*nadA*	582 bp fragment encoding quinolinate synthetase	nadAFw 5'-GCGCACAACTATCAGACACCGGAGAT-3' nadARw 5'-GCGACATTGTCGCTCATCGAGCATT-3'	DQ521662
	*minD*	589 bp fragment encoding septum site-determining protein	minDFw 5'-ATGATGGGGAAAGTGATCGTCGTCACGTC-3' minDRw 5'-CGAGCAGCGGGATGGACAGG-3'	JF920043
	*hutI*	577 bp fragment encoding imidazolonepropionase protein	hutIFw 5'-CGGCGGCGGCATCGTCTCCT-3' hutIRw 5'-CCACCGGCGGCTTCTGCTTTTCAT-3'	JF920044
	*pcaG*	344 bp fragment encoding protocatechuate 3,4-dioxygenase protein	pcaGFw 5'-CGGCGTCGCGATGGTCAA-3' pcaGRw 5'-CGGCGTTGGCCTCCGTCTC-3'	JF920045
	*repAb*	1309 bp fragment encoding replication/partition protein of pRleTA1b	repAbFw 5'-ATGCGGATCGTGCTGTCGTAGA-3' repAbRw 5'-GCCGCGGCCAACTCCTG-3'	FJ592235
	*repCb*	932 bp fragment encoding putative replication protein of pRleTA1b	repCbFw 5'-GGGAGCGCCTGACACTTTGCC-3' repCbRw 5'-GGAAGCAGGGTTTGAAGCATCGTA-3'	FJ592235

pRleTA1a	*nodA*	662 bp encoding fragment of acyltransferase nodulation protein	nodA-1 5'-TGCRGTGGAARNTRNNCTGGGAAA-3' nodA-2 5'-GGNCCGTCRTCRAAWGTCARGTA-3'	AY904443
	*nifNE*	649 bp fragment encoding nitrogenase MoFe cofactor biosynthesis proteins	NifNFw 5'-CCGGTCGGCGCATCTGTTCC-3' NifNRw 5'-GGTGCGCTGCCAATACTCCAT-3'	DQ471906
	*thiC*	478 bp encoding fragment of thiamine biosynthesis protein	thi3 5'-GGCCGGGGTTTTCGCGGATGGCGA-3' thi5 5'-TTCCGGCTGAGGACTGGGTCTCCAAT-3'	DQ535897
	*acdS*	890 bp encoding fragment 1-aminocyclopropane-1-carboxylate deaminase	acdSFw 5'-GTTCGAACGCTACCCGCTCACCTT-3' acdSRw 5'-TCCCCTGCATCGACTTTCCCTCAT-3'	EU700492
	*repAa*	774 bp fragment encoding replication/partition protein of pRleTA1a	repAaFw 5'-TCCTYYGCTGCGAAGATTACACG-3' repAaRw 5'-CGCGCCCAGGGTCAGATAGC-3'	HM032068
	*repCa*	773 bp fragment encoding putative replication protein of pRleTA1a	repCaFw 5'-GGGATYGCCGCCTACCGTGACAGT-3' repCaRw 5'-GCATAWCGGCCGACCCTCCCTACA-3'	HM032068

^a ^-ch-chromosome

### Preparation of high molecular weight DNA and PFGE conditions

The plugs were formed with 5 ml 48 h culture of *Rlt *strains, which after centrifugation were resuspended in TE buffer and mixed with 2% LMP agarose (Sigma). Agarose embedded cells were incubated with TE and lysozyme (1.5 mg/ml) for 16 h at 37°C, and then in cell lysis buffer (1% sodium lauryl sarcosine, 50 mM EDTA, 50 mM Tris-HCl pH 8.0) supplemented with proteinase K (0.5 mg/ml) at 37°C for additional 48 h. The proteinase K was inactivated by PMSF (0.4 mg/ml) at 37°C for 1 h. Plugs were washed tree times (30 min) with TE buffer and finally stored in TE at 4°C. PFGE was performed with the contour-clamped homogenous electric field mode with the Bio-Rad system (model CHEF-DRIII). DNA samples were separated in 1% Megabase agarose gels (Bio-Rad) in 1 × TAE buffer, refrigerated at 12-14°C, with switch time 100-300 seconds, angle 106°, voltage gradient 3 V/cm for 48 h. Estimation of plasmid size was performed with BIO-PROFIL BioGene (Vilber-Lourmat, France), using *R. leguminosarum *bv. *viciae *strain 3841 [6], *R. leguminosarum *bv. *trifolii *TA1 [25,26] and *Sinorhizobium meliloti *1021 [4].

### Computer assisted analyses

Sequence data were analyzed with Lasergene analysis software (DNASTAR, Inc). Data base searches were done with the BLAST and FASTA programs at the National Centre for Biotechnology Information (Bethesda, Md) and European Bioinformatic Institute (Hinxton, UK). For the DNA sequences multiple alignments Clustal-W algorithm was used [27]. Codon usage of sequenced genes was calculated using ACUA [28]. Codon adaptation index (CAI) was calculated with cai program [29]. In codon usage discriminant analyses with two grouping methods were applied to studied sequences: (a) based on the localization of genes in defined part of the rhizobial genome (three groups: chromosome, chromid-like, and other plasmids), or (b) based on the origin of the genes (13 groups-each for one strain). The results of this multivariate analysis give us the information about separation of studied groups on the basis of discriminant functions i.e. linear combinations of studied variables maximizing distances between groups and orthogonal to each other [30].

For every grouping method set of variables included the relative frequency of alternative codons (for the same aminoacids), leading to the investigation of 59 variables (omitting stop codons and codons for methionine and tryptophan, which have no alternatives).

Complete discriminant analysis was performed but from among many obtained results we focused on Chi-squared test providing the number of statistically significant discriminant functions, squared Mahalanobis distances between the group centroids (taking into account the correlation between variables), scatterplots of discriminant scores i.e. cases located in the property space formed by first two discriminant functions [31] as well as the classification table containing information about the number and percent of correctly classified cases in each group.

The application of discriminant analysis was preceded by tolerance test, which enable us to remove redundant variables out of the model [32]. The tolerance tests were performed using Classify/Discriminant unit of SPSS software (SPSS for Windows version 10.0, 1999, SPSS Inc., Chicago, IL, USA) while other results were obtained using Discriminant Function Analysis units of STATISTICA software system (Statistica version 6, 2001, StatSoft Inc., Tulsa, OK, USA).

### Nucleotide sequence accession numbers

The following GenBank accession numbers were given to the nucleotide sequences determined in this study. For *dnaC *GQ374266-GQ374277, *dnaK *GQ374278-GQ374289, *exoR *GQ374290-GQ374301, *fixGH *GQ374302-GQ374313, *hlyD *GQ374314-GQ374325, *lpsB *GQ374326-GQ374337, *nadA *GQ374338-GQ374349, *nifNE *GQ374350-GQ374361, *nodA *GQ374362-GQ374373, *prc *GQ374374-GQ374385, *rpoH2 *GQ374386-GQ374397, *thiC *GQ374398-GQ374409, *minD *JF920043, *hutI *JF920044, *pcaG *JF920045

## Results

### Strain selection based on variable genomic organization

**Figure 1:**
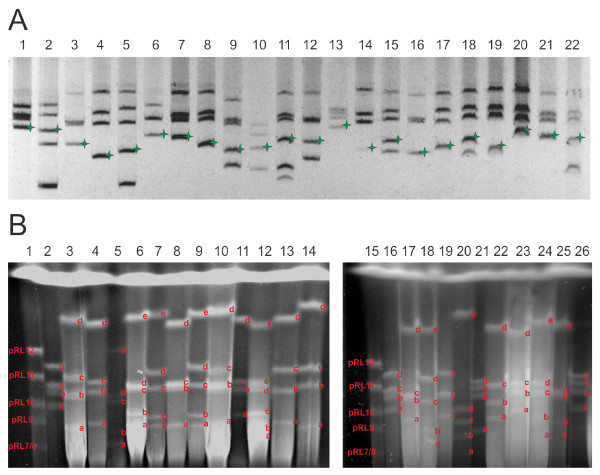
**Plasmid profiles of selected *R. leguminosarum *bv. *trifolii *nodule isolates**. (A) Profiles obtained in Eckhardt-type agarose gel electrophoresis; stars colored in green indicate pSym plasmids. Lanes: 1-*Rt*TA1; 2-*Rlv *3841; 3-K2.2; 4-K2.4; 5-K2.9; 6-K3.6; 7-K3.8; 8-K3.12; 9-K3.16; 10-K3.22; 11-K4.11; 12-K4.13; 13-K4.15; 14-K4.16; 15-K4.17; 16-K5.6; 17-K8.7; 18-K9.2; 19-K9.8; 20-K10.7; 21-K10.8, 22-K12.5 (B) PFGE separated replicons of *Rlt *nodule isolates further submitted to hybridization assays. The names of plasmids of *Rlv *3841 strain, used as molecular weight markers were shown [6]. Molecular weight of *Rlv *3841 plasmids is: 870, 684, 488, 353, 152, 147.5 kb. The letters on the respective bands of particular plasmids of individual strains indicates the plasmid name, e.g., "a" indicates pRlea plasmid. Lanes: 1-*Rlv 3841*; 2-*RtTA1*; 3-K2.4; 4-K3.12; 5-K3.16; 6-K4.13; 7-K4.17; 8-K5.6; 9-K9.2; 10-K10.4; 11-K3.8; 12-K4.11; 13-K8.7; 14-K9.8; 15-*Rlv *3841; 16-*Rt*TA1; 17-K2.2; 18-K2.9; 19-K3.6; 20-K3.22; 21-K5.4, 22-K10.7, 23-K10.8, 25-K3.13, 26-K4.15.

A group of 23 isolates was selected from among a collection of 129 *R. leguminosarum *bv. *trifolii *(*Rlt*) isolates recovered from nodules of ten clover plants grown in the vicinity of each other in cultivated soil. The main criterion of strain selection, beside the ability of effective nodulation of clover (*Trifolium pratense*), was their different plasmid pattern obtained by Eckhardt's lysis procedure (Figure 1A). The strains harbored from 3 to 6 plasmids whose size, as assessed by PFGE analysis of high molecular weight (HMW) genomic DNA, ranged approximately from 150 kb to 1380 kb (Table 2, Figure 1B). The plasmids will be referred to as pRlea to pRlef throughout this report. The isolates that differed in the plasmid pattern were assumed to be distinct strains. In all the strains studied, the single symbiotic plasmid (pSym), with average molecular weight of 361 kb (ranging from 260 kb to 500 kb) was identified by Southern hybridization with *nodA *and *nifNE *probes, derived from the *R. leguminosarum *bv. *trifolii *TA1 (*Rt*TA1) laboratory strain [26]. A set of 24 strains (including *Rt*TA1) with a highly variable number and size of plasmids was chosen for further hybridization assays. Noteworthy is the presence of very large plasmids with molecular weight above 1.0 Mb, identified in a majority of the sampled strains (Figure 1).

**Table 2:** Plasmid number and size of *R. leguminosarum *bv. *trifolii *strains determined by PFGE

*Rlt *strains	Plasmid size (kb)					
	
	pRlef	pRlee	pRled	pRlec	pRleb	pRlea
*Rt*TA1			808	653	603	476*
K3.8			1110	640	570	370*
K3.13		1210	610	590	350*	240
K3.16		915	570	520	270*	200
K3.22		1350	510	420	310*	185
K8.7			1110	710	560	330*
K9.8			1250	710	580	260*
K10.7			1180	710	565	430*
K10.8			1120	670	600	460*
K12.5		1220	670	580	395*	270
K3.6				840	620	430*
K4.11	1060	610	560	350*	190	150
K4.15			770	705	640	500*
K2.2			1230	650	630	440*
K2.4			1250	720	570	320*
K4.13		1240	650	630	420*	310
K4.16			1380	680	585	320*
K4.17		1140	700	600	330*	250
K5.4			780	690	650	335*
K9.2		1140	730	620	340*	250
K10.4			1130	700	570	290*
K2.9		1240	810	590	375*	180
K3.12			1210	700	630	400*
K5.6			1060	635	610	290*

*-symbiotic plasmids

Average molecular weight (m.w.) of all the plasmids in each of the 23 isolates was calculated as 2.815 Mb (ranging from 1.89 to 3.25 Mb). With regard to the average genome size ~7.145 Mb of recently sequenced *R. leguminosarum *bv. *trifolii *WSM2304 (*Rlt*2304) and WSM1325 (*Rlt*1325) [33,34], in which extrachromosomal replicons constitute 34% and 36%, respectively, the extrachromosomal DNA content in our strains was calculated to range from 26% to 45% (an average ~39%).

### Similarity of replication-partition genes in the plasmid pool of selected strains

One of the methods to assess the phylogenetic relatedness among plasmids is to compare their replication systems. Thus, at the beginning of our study, similarity and/or diversity of replication regions between the plasmids of the nodule isolates were examined. Recently, the replication systems of four plasmids (pRleTA1a-pRleTA1d), each equipped with *repABC *genes, were analyzed in *Rt*TA1 [35]. An experimental approach comprising a series of Southern hybridizations with *repA *and *repC *genes derived from plasmids pRleTA1a-pRleTA1d of *Rt*TA1 as molecular probes was used (Table 1). The *repA *and *repC *genes were PCR amplified from the *Rt*TA1 genome and probed against PFGE-separated HMW DNA of the sampled strains. The choice of two different genes from each of the replication system identified in *Rt*TA1 as molecular probes seemed to be justified by lack of single universal phylogenetic history within the *repABC *operon and by RepA and RepB evolution, partially independent from RepC [13].

**Figure 2:**
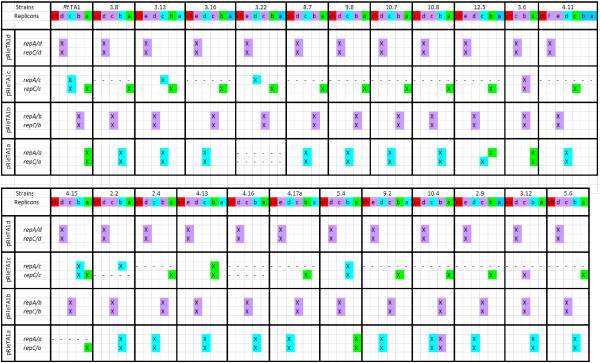
**Replication/partition gene distributions in the tested *Rlt *nodule isolates**. Southern hybridization assays were carried out with *repA *and *repC *markers of defined *Rt*TA1 plasmids as molecular probes. The position of given markers in *Rt*TA1 genome was shown in the left column. Positive hybridization was colored regarding its location in one of the following genome compartments: chromosome (red), plasmids (blue) and pSym (green); (-) indicates that given marker was not detected within a genome under applied Southern hybridization conditions. The letters a-f below the strains name indicate respective plasmids, ch-chromosome.

Distribution of the given *rep *marker was assessed with regard to its location in one of the extrachromosomal replicons of the tested strains. *repA *and *repC *genes of the largest pRleTA1d were jointly detected on the largest plasmids in all the sampled *Rlt *strains (Figure 2). Similarly, *repA *and *repC *of the pRleTA1b jointly hybridized to one of the plasmids of different size in all the *Rlt *strains. In contrast, *repA *and *repC *of the pRleTA1c were rarely localized together (4 of 23 strains). The *repA *of the pRleTA1c was not similar to any of the plasmids in most of the sampled strains, but *repC *hybridized frequently (19 of 23 strains) to pSym plasmids. *repA *and *repC *of pRleTA1a (pSym) commonly showed sequence similarity to non-symbiotic plasmids of the sampled strains and only exceptionally hybridized to symbiotic ones (Figure 2).

RepABC of pRleTA1d and pRleTA1b display similarity with replication systems of the extrachromosomal replicons, which were recently described as chromids [16,35]. Within the group of closely related strains *Rt*TA1, *R. leguminosarum *bv. *viciae *3841 (*Rlv*), *R. etli *CFN42 (*Rhe*), *Rlt*WSM2304 and *Rlt*WSM1325 clusters of replicons carrying the most similar replication systems can be distinguished. They comprise pRleTA1d-pRL12-p42f-pRLG201-pR132501 and pRleTA1b-pRL11-p42e-pRLG202-pR132502, respectively. Therefore, detection of positive hybridization signals with probes derived from *rep *genes of *Rt*TA1 chromid-like replicons (i.e. pRleTA1b or pRleTA1d) to any of the replicons of the sampled strains allowed regarding those as a chromid-like. Based on the similarity of replication-partition genes detected in our assays, we divided the replicons of the studied strains into three genome compartments: chromosome, chromid-like and 'other plasmids' (i.e. those replicons which gave a hybridization signal with molecular probes originating from *repA *and *repC *genes of pRleTA1a or pRleTA1c, as well as those that gave no signal with any *rep *probes of *Rt*TA1 replication genes). The compartment designated 'other plasmids' also comprised pSym. Such replicon division was taken into consideration in the subsequent analyses of distribution of other markers in the studied strains.

### Variability of chromosomal and plasmid marker location

In further studies, the extent of gene content diversity in the sampled nodule isolates was examined. We aimed to estimate whether, besides *repA *and *repC *displacement events, we could demonstrate changes in the location of the chromosomal and plasmid genes. The same experimental approach was used, i.e. a series of Southern hybridizations with different genes with a well-defined chromosomal or plasmid location in *Rt*TA1 (Table 1) [36].

For assays of chromosomal marker variability, essential bacterial genes were chosen: *rpoH2*, *dnaK*, *dnaC, rrn*, *lpxQ *as well as genes that are not essential or with unspecified essentiality but chromosomal in *Rt*TA1, i.e. *bioA*, *stbB*, *exoR*, *pssL *(Pss-I) and *rfbADBC *(Pss-V) (Table 1). In addition, location of *fixGH *genes was assayed, even though they are known to be plasmid located on the sequenced *Rlt*WSM2304, *Rlt*WSM1325 [33,34], *Rlv *[6] and *Rhe *[5] genomes, but chromosomal in *Rt*TA1 [36].

**Figure 3:**
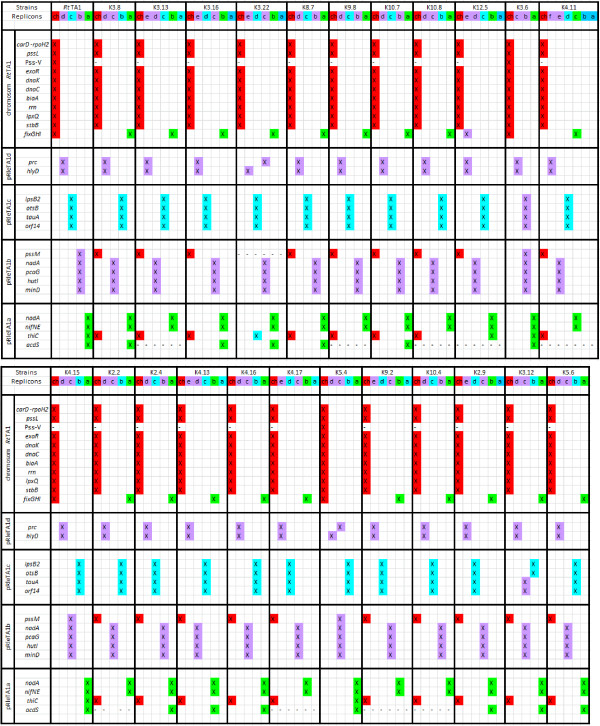
**Distribution of replicon specific genes in the tested *Rlt *nodule isolates**. Southern hybridization assays were carried out with several chromosome and plasmid markers of *Rt*TA1 as molecular probes. The position of a given markers in *Rt*TA1 genome was shown in the left column. Positive hybridization was colored regarding its location in one of the following genome compartments of *Rlt *isolates: chromosome (red), chromid-like (violet), plasmids (blue) and pSym (green); (-) indicates that given marker was not detected within a genome under applied Southern hybridization conditions. The letters a-f below the strains name indicate respective plasmids, ch-chromosome.

A majority of the studied genes (*rpoH2*, *dnaK*, *dnaC, rrn*, *lpxQ, bioA*, *stbB, exoR *and *pssL*) were located on the chromosome in all the sampled strains, showing considerable conservation of chromosomal markers (Figure 3). Exceptionally, the Pss-V region was identified on the chromosome of the K3.6, K5.4 and *Rt*TA1 but it was missing in the other strains (Figure 3) Moreover, *fixGH *symbiosis-related genes, which were chromosomal in the *Rt*TA1, K3.6, K4.15 and K5.4 strains, were located mainly in the genome compartment designated as 'other plasmids' (pSym to be exact) in the remaining strains. The variable location of *fixGH *genes which were found on the chromosome, pSyms and chromid-like replicons (K12.5) could be accounted for by location of these genes on the putative genomic island flanked by 18 bp repeats in *R. leguminosarum *and *R. etli *[10,37].

Southern hybridizations with probes comprising markers previously identified on different *Rt*TA1 replicons [36], such as *prc *and *hlyD *of pRleTA1d; *lpsB2*, *orf16*-*orf17-otsB*, *tauA *and *orf14 *genes cluster of pRleTA1c; *nadA *and *pssM *(surface polysaccharide synthesis region Pss-III) of pRleTA1b, were carried out. These analyses demonstrated that pRleTA1d markers were almost always jointly detected in the largest chromid-like replicons (only in K3.22 and K5.4 they are separated between distinct chromid-like replicons). pRleTA1c markers in almost all (21 out of 23) of the sampled strains were located in the genome compartment designated as 'other plasmids' (Figure 3). From among markers of pRleTA1b, *nadA, minD*, *hutI *and *pcaG *had always chromid-like location, while the *pssM *gene was located in the chromosome of 19 strains, in chromid-like replicons of four strains including *Rt*TA1, and was absent in the genome of K3.22 strain, respectively (Figure 3).

Besides the symbiotic genes *nodA *and *nifNE *used for identification of pSym plasmids, stability of *thiC *and *acdS *(Table 1) of the pRleTA1a symbiotic plasmid (*ipso facto *described as markers of the 'other plasmids' pool) was examined (Figure 3). Only *thiC *was identified in all the strains, however, located in different genomic compartments: most frequently on the chromosome (18 of 23 strains), and in the 'other plasmids' (5 strains). The *acdS *gene was detected in 14 of 23 strains, in each case on pSym (Figure 3). The *thiC *gene, similarly to *fixGHI*, showed high variability in location; however, its putative mobile element location is unknown [38]. *thiC *was reported as plasmid located in sequenced genomes of *Rlv *[6], *Rlt*2304 [33] and *Rhe *[5].

**Figure 4:**
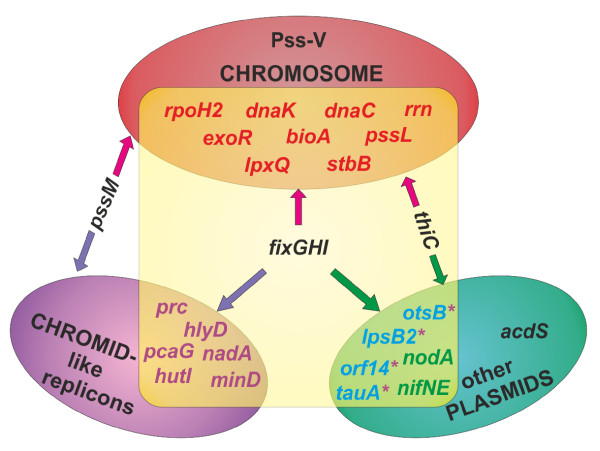
**Overall genes distribution in three genome compartments: chromosome, chromid-like and 'other plasmids' in *Rlt *isolates**. Southern hybridizations were carried out with *Rt*TA1 markers of specified localization as probes. The arrows indicate instability of some markers location in the given genome compartments. Asterisk indicates genes exceptionally localized on chromid-like replicon. Yellow area indicates genes detected in all tested strains.

As a result, genes with a stable location in specific genome compartments in all the strains, as well as unstable genes with variable, strain-dependent distribution were distinguished (Figure 4). Stable markers for each compartment of the sampled strains were established i.e. chromosomal: *rpoH2*, *exoR*, *dnaK*, *dnaC, bioA*, *rrn*, *lpxQ, pssL *and *stbB*; chromid-like: *prc, hlyD*, *nadA, minD*, *hutI *and *pcaG*; 'other plasmids': *otsB*, *lpsB2 *(exceptionally chromid-like in K3.6), *tauA *and *orf14 *(exceptionally chromid-like in K3.12) including *nodA *and *nifNE *symbiosis-related genes of pSym (Figure 4). Loss of some of the examined markers was noticed, i.e. Pss-V from the chromosome, *pssM *from chromid-like replicons, and *acdS *from the 'other plasmids' (pSym). Only two of the sampled strains, i.e. K3.6 and K5.4, contained all the studied markers, while others lacked at least one of the genes.

**Figure 5:**
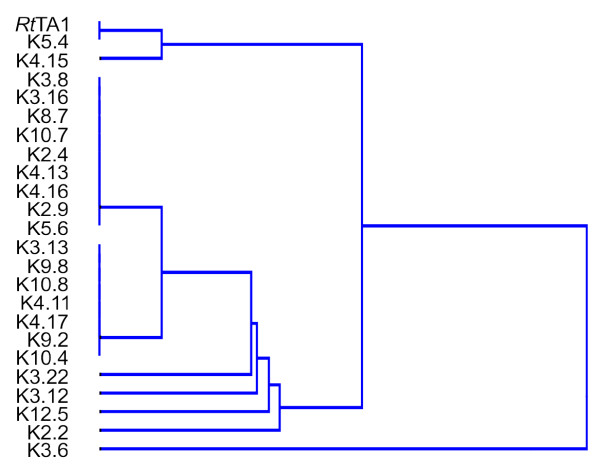
**The dendrogram showing similarity of *Rlt *nodule isolates and *Rt*TA1 strain**. The dendrogram was constructed on the basis of marker distribution among different genome compartments using UPGMA clustering method.

A dendrogram demonstrating similarity of the strains was constructed with the UPGMA clustering method based on markers distribution among their different genome compartments. It showed one K3.6 strain apparently split from the others (Figure 5), and two groups of clustered strains: a small one, including *Rt*TA1, K5.4 and K4.15, and a large one comprising the remaining strains, which was further subdivided into two smaller subgroups of strains with identical marker distribution (Figure 5).

### Sequence divergence of chromosomal and plasmid genes

**Figure 6:**
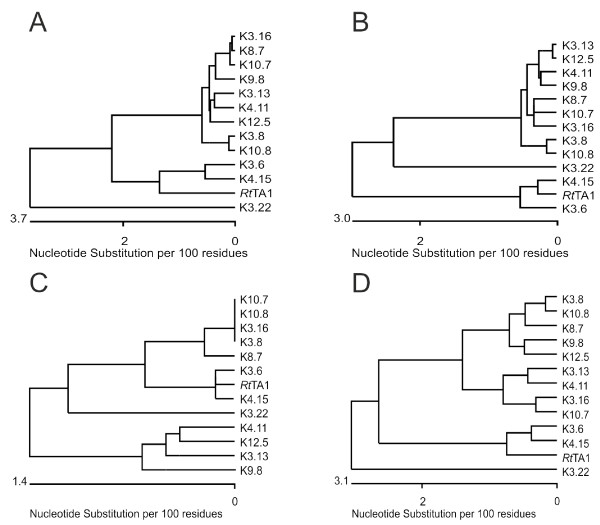
**The sequence similarity dendrograms of *Rlt *nodule isolates and *Rt*TA1 strain**. The dendrograms were constructed with UPGMA clustering method based on the chosen sequences of the given genome compartment: (A) concatenated chromosomal gene sequences; (B) chromid-like replicons'genes; (C) 'other plasmids' genes; (D) all gene sequences (stable and unstable) located in different genome compartments.

To assess the overall phylogenetic similarity of the sampled strains, several genes from a subset of 12 different strains displaying divergent plasmid profiles (plus *Rt*TA1) were partially sequenced and analyzed. The sequenced genes comprised exclusively chromosomal (*dnaC, dnaK, exoR, rpoH2*), chromid-like replicons (*hlyD, prc, nadA*), and 'other plasmid' markers (*nodA, nifNE*) as well as those with unstable location found in different genome compartments (*fixGH, thiC, lpsB2*). Afterwards, phylogenetic trees were constructed based on concatenated sequences of a distinct genome compartment, allowing description of the genetic similarity of the strains using the multilocus sequences analyses (MLSA) approach (Figure 6).

In general, a low number of nucleotide substitutions were found in the examined genes in most strains. Similar groups of clustered strains were obtained in dendrograms constructed both on the basis of concatenated chromosomal sequences (Figure 6A), as well as concatenated chromid-like replicon genes (Figure 6B). In both cases, a smaller group containing *Rt*TA1, K4.15 and K3.6 strains, and a larger group consisting of the remaining strains was observed. Interestingly, K3.22 chromosomal genes split off from all remaining strains suggesting their considerable divergence (Figure 6B). Sequence similarity within the *Rt*TA1, K4.15 and K3.6 group is also visible on a dendrogram exclusively based on plasmid gene sequences, derived from pSym (Figure 6C). When all the concatenated sequences (comprising genes with stable and unstable location in the genome) were used in dendrogram construction, the grouping of the strains was very similar to that obtained on the basis of stable chromosomal markers (Figure 6A, D). In conclusion, quite a similar phylogenetic history of the studied strains was demonstrated based on both stable and unstable chromosomal, chromid-like as well as 'other plasmid' genes (despite the small number of the markers analyzed).

**Figure 7:**
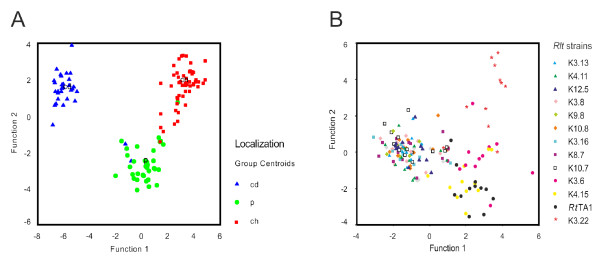
**Markers grouping obtained in discrimination analyses**. (A) Grouping was carried out regarding frequency of alternative codon usage. Symbols used: red squares-chromosome markers (ch), blue triangles-chromid-like replicons' markers (cd), green circles-'other plasmid' markers (including pSym markers) (p). (B) Strains grouping observed in discrimination analyses regarding frequency of alternative codon usage of the tested gene set.

To further evaluate the degree of sequence differentiation between the alleles with respect to their distribution in the genome and *eo ipso *the rate of adaptation to the genome compartment, we performed discrimination analyses focused on alternative codon usage. Discrimination analysis was applied to 59 variables (all potential triplets except for stop and non-alternative codons Met, Trp). Genes belonging to the chromosome, chromid-like and 'other plasmids' differed substantially with respect to this parameter (Figure 7A). Apart from the well-separated sequences belonging to the three distinct genome compartments, one can observe a subgroup localized between chromosomal and 'other plasmids' gene pools (Figure 7A). This subgroup comprised genes *thiC, fixGH*, which frequently changed their location and their codon usage was not adapted to any genome compartment. Comparison of the results of gene grouping based on hybridization data and discrimination analysis demonstrated very high accordance equal to 96%.

The discrimination analysis of codon usage performed on individual strains harboring the set of the tested genes (13 groups of sequences) revealed only minor differences between the resultant groups and almost no accordance (31%) with the grouping performed on the basis of hybridization. However, some level of similarity between the strains can be demonstrated. As a consequence, one more discrimination analysis of codon usage was done, and the strains were divided into three groups: (i) K3.22, (ii) *Rt*TA1, K3.6, K4.15 and (iii) all the remaining strains (Figure 7B). This resulted in 92% accordance between codon usage-based and strain-dependent grouping of sequences (Figure 7B and Figure 6D). It was concluded that codon usage was not significantly influenced by the individual strains but may be characteristic for the group of strains.

Finally, the Codon Adaptation Index (CAI) of the sequences studied was calculated. The CAI can be used to "evaluate the extent to which selection has been effective in molding the pattern of codon usage" [29] as well as to compare the codon usage of foreign genes versus that of highly expressed native genes [13]. Here, we applied CAI analyses to assess the degree of adaptation of sequenced genes to the host by comparing the obtained CAI values with those of genes encoding ribosomal proteins in *R*. *leguminosarum*. The calculated CAI values for each sequence were arbitrarily grouped and subsequently submitted to ANOVA evaluation, which measures the significance of differences between groups. CAI values can range from 0 (reflecting use of synonymous codons) to 1 (reflecting the strongest bias where codon usage is equal to that in the ribosomal protein-encoding genes) [13].

The CAI values ranged from 0.849 (*dnaC*-chromosomal gene) to 0.554 (*nodA-*symbiotic gene). The *fixG *and *thiC *had the CAI equal to 0.676 and 0.673, respectively, suggesting weaker adaptation to their genome compartments and further confirming their unstable location as indicated in hybridization analyses. We did not find significant differences with respect to the CAI values calculated for the particular strains, but strains *Rt*TA1, K4.15, K3.6, and K3.22 previously observed as most divergent had a high average CAI of the studied sequences (from 0.722 to 0.718), possibly indicating good adaptation of the genes to the host. Finally, the CAI values were evaluated according to the location of genes in the different genome compartments (Table 3). The CAI values of genes located on the chromosome and chromid-like replicons were high and significantly differed from each other. The genes located on the 'other plasmids' (including pSym) had the lowest CAI values significantly different from the former ones. These results demonstrated weaker adaptation of plasmid genes to the host genome in comparison to the chromosome and chromid-like genes.

**Table 3:** The Codon Adaptation Index (CAI) of genes located in genome compartments in *Rlt *nodule isolates.

Gene location	Number of sequences	Average CAI
Chromosome	66	0.767 ± 0.062 ^a^

Chromid-like	42	0.732 ± 0.065 ^b^

Other plasmids	61	0.645 ± 0.061 ^cd^

Values followed by the various letters are significantly different: ^b ^(*P *< 0.05) and ^cd ^P < 0.001

± Standard deviation (SD)

## Discussion

Three genome compartments that differed genetically and functionally can be distinguished in the nodule population of *R. leguminosarum *bv. *trifolii*: the chromosome, chromid-like and 'other plasmids' including pSym. Chromid-like replicons were distinguished in Southern analyses on the basis of *repA *and *repC *sequence similarity to *Rt*TA1 and to the respective replication genes of such replicons described in the sequenced genomes of *R. leguminosarum *bv. *viciae*, *R. etli *and *R. leguminosarum *bv. *trifolii *[16]. The chosen name "chromid-like" (as opposed to simply "chromid") was the result of data scarcity concerning their gene content, insufficient to justify the name "chromid" [16]. Moreover, it is known that genes of the *repABC *operon are peculiar genetic markers because of the complex phylogeny of particular genes within the operon, whose evolutionary history could not be strictly connected with other genes of particular replicons [13].

In the study of the distribution of several chromosomal and plasmid markers within a group of 23 nodule isolates, stable genes permanently located in a specific *R. leguminosarum *bv. *trifolii *genome compartment: chromosome, chromid-like and 'other plasmids' including pSym were distinguished. Unstable genes (*fixGH*, *thiC*, *acdS*, *pssM *and Pss-V region) that changed their location at various rates or were lost from the genome were also detected. Only two of the sampled 23 strains possessed all the studied markers. A majority of strains differed in the gene content and gene distribution, supporting the hypothesis of the pangenomic structure of *R. leguminosarum*, in which each strain of a given species contains, besides the core genome, additional genetic information specific for the strain [11,17,18,39].

The distribution of the plasmid replication-partition genes was even more dynamic than that of genes not connected with replication. Independent transfer events of *repA *and *repC *genes of the putative *repABC *operon were frequently observed, especially in the 'other plasmids' compartment, which confirmed different evolutionary pathways for various elements of the *repABC *operon, recently evidenced in *Alphaproteobacteria *[13]. Such considerable dynamics of replication/partition gene distribution in *Rhizobium *may account for changes in the plasmid number and, consequently, gene content observed in the sampled population. Beside the dynamics of replication/partition gene distribution, some level of conservation of replication genes, especially those of chromid-like replicons, was also observed. It was reflected in positive hybridizations with pRleTA1d and pRleTA1b derived *rep *probes, to the respective replicons of *Rlt *strains. One could speculate that the conservation of replication genes of chromid-like replicons may be related with their distinct properties e.g. stability. However, the gene content rather than the properties of the replication system, resulting e.g. from conservation of replication genes, seem to be crucial for replicon stability [40].

Redistribution of genes between the different genome compartments could further trigger their sequence divergence under different selective pressures [13,15,41]. Examination of sequence divergence of several stable and unstable chromosomal and plasmid genes showed a low level of substitutions in genes of all the compartments. Nearly identical nucleotide sequences of *nifNE *markers were found in different pSym plasmids of the studied population (Figure 6C), confirming the core character of symbiotic genes and their high conservation, despite the overall genome differentiation [11].

The extent of gene adaptation to a given compartment in the host genome was assessed by analyses of alternative codon usage. Three groups of well separated genes were obtained corresponding to the chromosome, chromid-like and 'other plasmids' genome compartments (Figure 7A) with 96% accordance with hybridization data. In conclusion, the sequence divergence of particular genes may be affected by their location in the given genome compartment. When all the sequences of the individual strains studied were subjected to a discrimination analysis, we obtained good separation of K3.22 and a group of strains related to *Rt*TA1 (Figure 7B) that formed the outermost branch in the phylogenic tree. The remaining strains were randomly mixed with each other but apparently separated from K3.22 and TA1-related strains, which suggested no differences in codon usage within the main group.

The CAI analyses of the evaluated sequences confirmed good adaptation of chromosomal and chromid-like genes (high CAI values) to host genomes and lower CAI values for 'other plasmids' genes. The CAI values also reflect the level of transcriptional and translational activity of particular genes [29]. While the activity of most of the chromosomal and chromid-like genes could be considered at least to some extent constitutive, the 'other plasmids' and especially symbiosis-related genes are expressed only transiently in the symbiotic stage [42]. Therefore, in the *Rhizobium *model, the differences in codon usage in translation reflect the balance between the selection pressure and random mutations in the functionally differentiated genome compartments. The differences in codon usage and CAI values between the genome compartments are most likely a consequence of differential gene expression and adaptability to optimal codon usage in host genomes [42].

## Conclusion

Our study showed that, even within a small rhizobial population of clover nodule isolates, substantial divergence of genome organization can be detected especially taking into account the content of extrachromosomal DNA. Despite the high variability with regard to the number and size of plasmids among the studied strains, conservation of the location as well as the dynamic distribution of the individual genes (especially replication genes) of a particular genome compartment was demonstrated. The sequence divergence of particular genes may be affected by their location in the given genome compartment. The 'other plasmid' genes are less adapted to the host genome than the chromosome and chromid-like genes.

## Authors' contributions

AM designed and coordinated the study and drafted the manuscript. GS conducted the bulk of the experiments: carried out analyses of *Rlt *plasmid content by Eckhardt' method and PFGE, prepared and labeled the molecular probes and provided Southern hybridization. JW helped in statistical analyses, contributed to interpretation of data and preparation of the manuscript. AKK performed the statistical analysis. MMK collected rhizobial strains and helped with *Rlt *plasmid analyses. AS provided scientific guidance and discussion and prepared final version of manuscript. All authors have read and approve of this final manuscript.
